# Single-molecular diffusivity and long jumps of large organic molecules: CoPc on Ag(100)

**DOI:** 10.3389/fchem.2024.1355350

**Published:** 2024-02-06

**Authors:** Agata Sabik, John Ellis, Holly Hedgeland, David J. Ward, Andrew P. Jardine, William Allison, Grażyna Antczak, Anton Tamtögl

**Affiliations:** ^1^ Institute of Experimental Physics, University of Wrocław, Wrocław, Poland; ^2^ Department of Semiconductor Materials Engineering, Wrocław University of Science and Technology, Wrocław, Poland; ^3^ Cavendish Laboratory, Cambridge, United Kingdom; ^4^ Institute of Experimental Physics, Graz University of Technology, Graz, Austria

**Keywords:** surface diffusion, energy dissipation, single-molecule studies, organic thin films, atom-surface sattering, friction

## Abstract

Energy dissipation and the transfer rate of adsorbed molecules do not only determine the rates of chemical reactions but are also a key factor that often dictates the growth of organic thin films. Here, we present a study of the surface dynamical motion of cobalt phthalocyanine (CoPc) on Ag(100) in reciprocal space based on the helium spin-echo technique in comparison with previous scanning tunnelling microscopy studies. It is found that the activation energy for lateral diffusion changes from 150 meV at 45–50 K to ≈100 meV at 250–350 K, and that the process goes from exclusively single jumps at low temperatures to predominantly long jumps at high temperatures. We thus illustrate that while the general diffusion mechanism remains similar, upon comparing the diffusion process over widely divergent time scales, indeed different jump distributions and a decrease of the effective diffusion barrier are found. Hence a precise molecular-level understanding of dynamical processes and thin film formation requires following the dynamics over the entire temperature scale relevant to the process. Furthermore, we determine the diffusion coefficient and the atomic-scale friction of CoPc and establish that the molecular motion on Ag(100) corresponds to a low friction scenario as a consequence of the additional molecular degrees of freedom.

## 1 Introduction

The self-assembly and growth of aromatic *π*-conjugated organic molecules adsorbed on metal surfaces is of paramount importance for the fabrication of molecular electronic devices. Controlling the growth of thin films composed of organic molecules implies understanding the interplay between the structure of the film and the dynamics of the molecules on the substrate from the early stages of film deposition. The initial stages of these phenomena involve mass transport where molecular species overcome the energy barrier between two (meta)stable adsorption configurations (Barth et al., 2005). Due to the complex nature of organic molecules, the translational motion on surfaces may be accompanied by rotations or conformational changes (Marbach and Steinruck, 2014). Moreover, for aromatic organic molecules, the interaction with the substrate often occurs via weak van der Waals (vdW) forces and friction may become so low that the molecule exhibits a diffusive motion in the ballistic regime (Calvo-Almazán et al., 2016). The variety of these processes and possible correlation between those makes the study of surface diffusion challenging (Ferrando and Jardine, 2020). In the traditional view of atomistic surface diffusion, the motion is realised by a random walk of the adatom by crossing a barrier between two neighbouring potential wells: Particles move by hopping from one favourable adsorption site to one of the nearest adjacent sites, following the energetically most favourable route across the potential energy surface (PES). This simple picture holds at low temperatures for a number of systems, specifically for self-diffusion of single atoms across transition metal surfaces (Antczak and Ehrlich, 2007; 2010), but becomes more complex for larger molecules as well as with increasing temperature.

**FIGURE 1:**
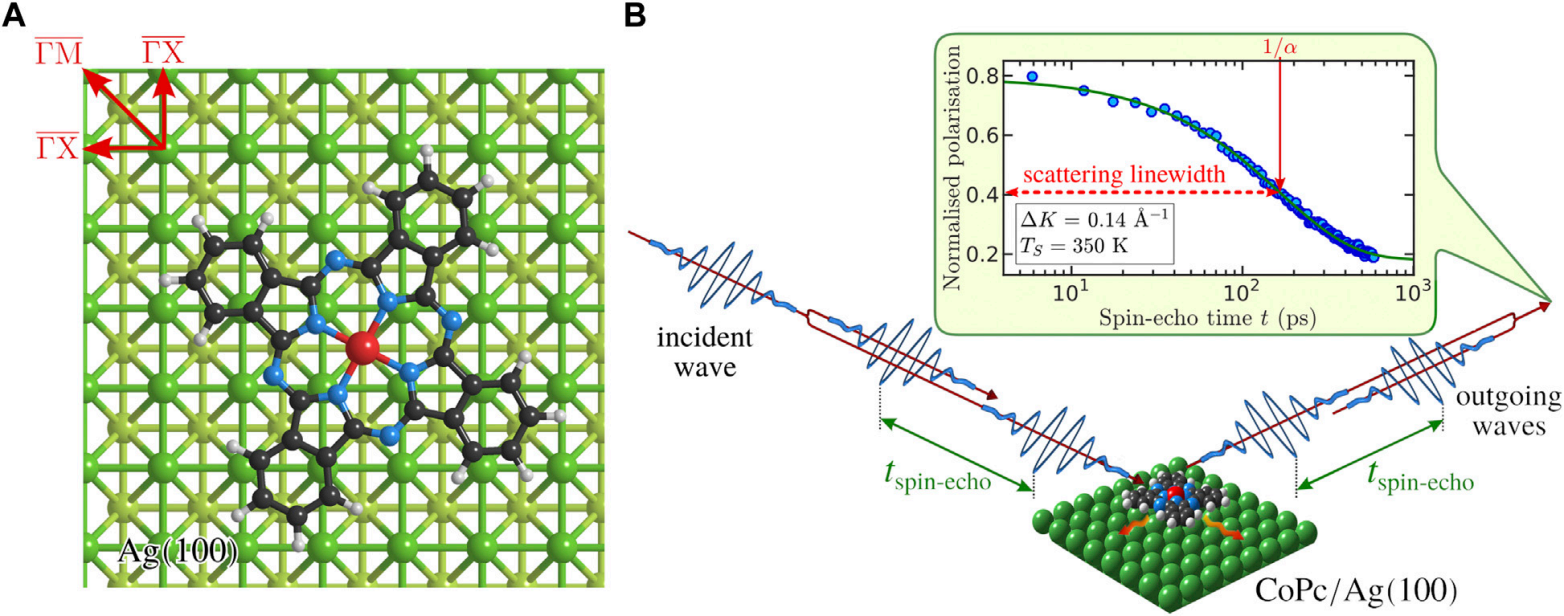
**(A)** Geometry of CoPc (C_32_H_16_CoN_8_) adsorbed on the top-most Ag(100) layer according to (Antczak et al., 2015a). The molecule adsorbs with the central Co atom located at the hollow site. The high-symmetry directions of the Ag(100) surface are illustrated as well. **(B)** Illustration of the helium spin-echo method where two wavepackets scatter with a time difference *t*_spin−echo_ from the surface, allowing the molecular motion to be interrogated through a loss in correlation, measured via beam polarisation. The top inset shows an exemplary intermediate scattering function (ISF) in terms of normalised polarisation *versus* spin-echo time *t* (filled circles) which is fitted with a single exponential decay (Eq. 1, solid green line) characterised by the dephasing rate *α* (scattering linewidth). The logarithmic time axis shows that a single exponential provides a good description of the experimental data.

Scanning tunnelling microscopy (STM) allows the observation of these molecular dynamics on surfaces and has been employed as a powerful tool to obtain quantitative information about thermally activated processes, i.e., surface diffusion (Weckesser et al., 1999; Weckesser et al., 2001; Schunack et al., 2002; Kwon et al., 2005; Eichberger et al., 2008; Buchner et al., 2011). From temperature-dependent measurements the activation energy for diffusion, the diffusivity prefactor, and the frequency prefactor or attempt frequency for the rate of barrier crossing can be determined. However, the temperature window accessible to STM investigations is strongly limited to a region where the frequency of molecular jumps is relatively slow in comparison to the time needed for a scan. Here we show that helium-spin echo (HeSE) spectroscopy allows measurements of molecular diffusion at a much higher temperature range. As illustrated in Figure 1B and further described in Section 2, lineshape broadening upon scattering a wave can be used to measure the motion of single molecules down to the relevant picosecond timescales at elevated temperatures. Using HeSE facilitates the collection of data complementary to the existing STM study, allowing measurements of diffusion in a much higher temperature range at coverages comparable to the STM data. The technique also permits measurements in the high coverage regime at elevated temperatures to enable a broader understanding of the adsorbate’s surface energetics and the role of molecule-molecule interactions.

The diffusion of phthalocyanine molecules (Pc, C_32_H_16_MN_8_, where M denotes a metal atom) on smooth metal surfaces, such as Ag(100) is one of the representative examples where STM was successfully employed to explore the basic surface diffusion steps. Pcs are good candidate molecules for organic semiconductors as described below and have thus been studied extensively in this context. The characteristic four-leaf feature/shape of the molecule as illustrated in Figure 1A, allows for tracking its diffusion in consecutive STM images (Antczak et al., 2015a). Pcs on Ag(100) are a good model system that allows both STM measurements at cryogenic temperatures and millisecond timescales as well as HeSE measurements at much higher temperatures and picosecond timescales. Most importantly, such a model system has to be stable over a wide temperature range, meaning that any thermal desorption or other thermally induced processes such as molecular decomposition or island creation have to be excluded over the studied temperatures (Kröger et al., 2010). Specifically, we study the motion of cobalt phthalocyanine (CoPc, C_32_H_16_CoN_8_) on Ag(100), where a combination of translational and rotational movements with an activation energy of 0.15 eV was found in STM measurements (Antczak et al., 2015a). However, due to the high mobility of CoPc, the investigated temperature interval was relatively narrow and covers the range from 45 to 50 K. Further, it was shown that above 75 K the molecular jumps are so frequent that it is impossible to recognise where the molecule is in an STM image (Antczak et al., 2017).

The family of Pc molecules is also considered as promising candidate for active layers in electronic devices, e.g., organic solar cells or organic field emission transistors (Wu et al., 2013; Melville et al., 2015) and recently the properties of Pc on monocrystalline metal surfaces have been widely studied to get a better insight into the performance of electronic devices (Gottfried, 2015). Pcs commonly adsorb with their aromatic plane parallel to the metal surface forming ordered structures, though for most metal-phthalocyanines no island formation is observed in the sub-monolayer regime at room temperature (Kröger et al., 2012; Feyer et al., 2014). CoPc studies have also included its utilisation as a single molecular magnet (Bartolomé et al., 2014; Wang et al., 2021) with possible applications in spintronics (Iacovita et al., 2008) and even quantum computing where the molecules act as single qubits (Warner et al., 2013; Bader et al., 2016). However, while a solid-state quantum computational architecture is likely to take the form of an ordered array assembled on a surface, recent studies have not considered this option (Graham et al., 2017). Finally, the adsorption of CoPc can also be used to manipulate the electronic surface states of a topological insulator (Caputo et al., 2016) and they have been successfully studied in the field of electrocatalysis for electrochemical CO_2_ reduction (Feng et al., 2022; Ding et al., 2023) and as possible gas sensors (Bohrer et al., 2007).

Our study illustrates broad agreement for molecular diffusion studied with two different experimental techniques in different temperature regions: STM data taken in real space at low temperature (Antczak et al., 2015a) and HeSE data taken in reciprocal space at a temperature seven times higher. While our results confirm the current understanding of a complex diffusion mechanism over a large temperature window, they illustrate that HeSE is able to observe subtle details such as the occurrence of long jumps at high temperatures and adsorbate interactions. As one may anticipate, there are indeed differences in the molecular motion such as a change of the effective diffusion barrier and the mentioned onset of long jumps with a distinctive motion of the molecules at high temperatures. Hence we show that by combination of STM with HeSE, it is possible to obtain a complete picture of complex diffusion mechanisms over the entire temperature range, from the onset of diffusion to technologically relevant temperatures.

## 2 Methods

The extension of the existing STM diffusion study of CoPc on Ag(100) to a higher temperature range requires methods that probe the nanometre length scale at an extremely short timescale. Very fast molecular dynamics has been successfully investigated by scattering techniques such as helium atom scattering (Tamtögl et al., 2021; Sacchi and Tamtögl, 2023) or neutron scattering (Tamtögl et al., 2018). The reported measurements were performed on the Cambridge helium-3 spin-echo (HeSE) apparatus where a nearly monochromatic beam of ^3^He is generated and scattered off the sample surface in a fixed 44.4° source-target-detector geometry. For a detailed description of the apparatus please refer to (Alexandrowicz and Jardine, 2007; Jardine et al., 2009; Holst et al., 2021). Prior to the deposition of CoPc, the Ag(100) substrate was cleaned by several Ar^+^ ion sputtering (0.8 keV energy, 10 μA sputtering current) and annealing cycles to 800 K. The quality and cleanliness of the crystal were checked with He diffraction measurements. CoPc was deposited using a home-built Knudsen cell which was filled with crystalline powder (Sigma-Aldrich) and kept at about 620 K while the Ag(100) sample was at 350 K.

Pcs arrange into well-ordered islands on smooth metal surfaces after the so-called critical coverage is reached. In the case of Ag(100) at room temperature, CoPc island creation is detected from around 0.8 monolayer (ML) onward (Wagner et al., 2022a; b). Between critical and ML coverage, the coexistence of mobile molecules in a so-called 2D gas phase with ordered islands was found by photoemission electron microscopy investigations (Wagner et al., 2022a). The molecular 2D gas phase manifests itself as irregular blurring or streaking in STM scan images since mobile molecules at increased sample temperatures result in noise detection by the STM tip (Antczak et al., 2017). The commonly reported superstructure associated with 1 ML of CoPc on Ag(100) is a (5 × 5) superstructure (Sabik et al., 2016). In general, Pcs adsorb with its aromatic plane parallel to flat metal surfaces, and the adsorption geometry of a single CoPc molecule on Ag(100) is presented in Figure 1A. The processes of adsorption can be observed by monitoring the specularly reflected helium signal while dosing CoPc onto the surface. The so-called uptake curve is shown in the Supplementary Material (Adsorption, uptake and coverage calibration). The deposited coverage can then be estimated based on the uptake curve where ML coverage corresponds to the (5 × 5) superstructure (Salomon et al., 2013; 2015; Sabik et al., 2016)). Using a helium scattering cross section of $\Sigma =1000\;{\overset{{}^{\circ}}{A}}^{2}$ for a single CoPc molecule on Ag(100) gives a coverage of 0.11 ML for a specular attenuation of *I*_0_/2. Most dynamics measurements were performed at this coverage unless otherwise stated (see Adsorption, uptake and coverage calibration in the Supplementary Material).

A helium spin-echo experiment as shown schematically in Figure 1B, provides direct access to the so-called intermediate scattering function (ISF), *I*(Δ**K**, *t*), at a fixed surface parallel momentum transfer Δ**K** specified by the scattering geometry. The measured beam polarisation is proportional to *I*(Δ**K**, *t*), which is related to the pair correlation function through a Fourier transform (Jardine et al., 2009; Tamtögl et al., 2020; Tamtögl et al., 2021; Sacchi and Tamtögl, 2023). As illustrated in Figure 1B, due to the motion of the adsorbate on the surface the (auto) correlation determined through the ISF decays with increasing spin-echo time, *t*. In its basic form, the time dependence follows a simple exponential decay:

$$I\left(\Delta K,t\right)=I_{0}\left(\Delta K,0\right)\cdot {\text{e}}^{-\alpha \left(\Delta K\right)\cdot t}+C\left(\Delta K\right)$$ (1)

with the decay constant (so-called dephasing rate) *α* and *I*_0_ the amplitude at *t* = 0. As can be seen in a typical ISF in the top inset of Figure 1B, the CoPc data is well represented by a single exponential decay. We note, however, that for other systems and different types of motion occurring on different timescales *I*(Δ**K**, *t*) will deviate from the simple form and is better fitted using multiple exponential decays (Jardine et al., 2009).

## 3 Results

We have performed HeSE measurements after the deposition of a specified amount of CoPc onto the clean Ag(100) substrate (see Section 2). The dynamics of CoPc adsorbed on Ag(100) were extracted from HeSE measurements, via the ISF, *I*(Δ**K**, *t*) with a single exponential decay according to Eq. 1. The timescales of molecular motion follow from the dephasing rate *α*, and the functional dependence of *α*(Δ**K**) *versus* momentum transfer Δ**K** provides details about the diffusive motion and the length scales of the latter in real space. The temperature dependence *α*(*T*) reveals the energetic diffusion barriers and can then be directly compared with low-temperature STM data taken in real space.

### 3.1 Jump diffusion of CoPc on Ag(100)

**FIGURE 2:**
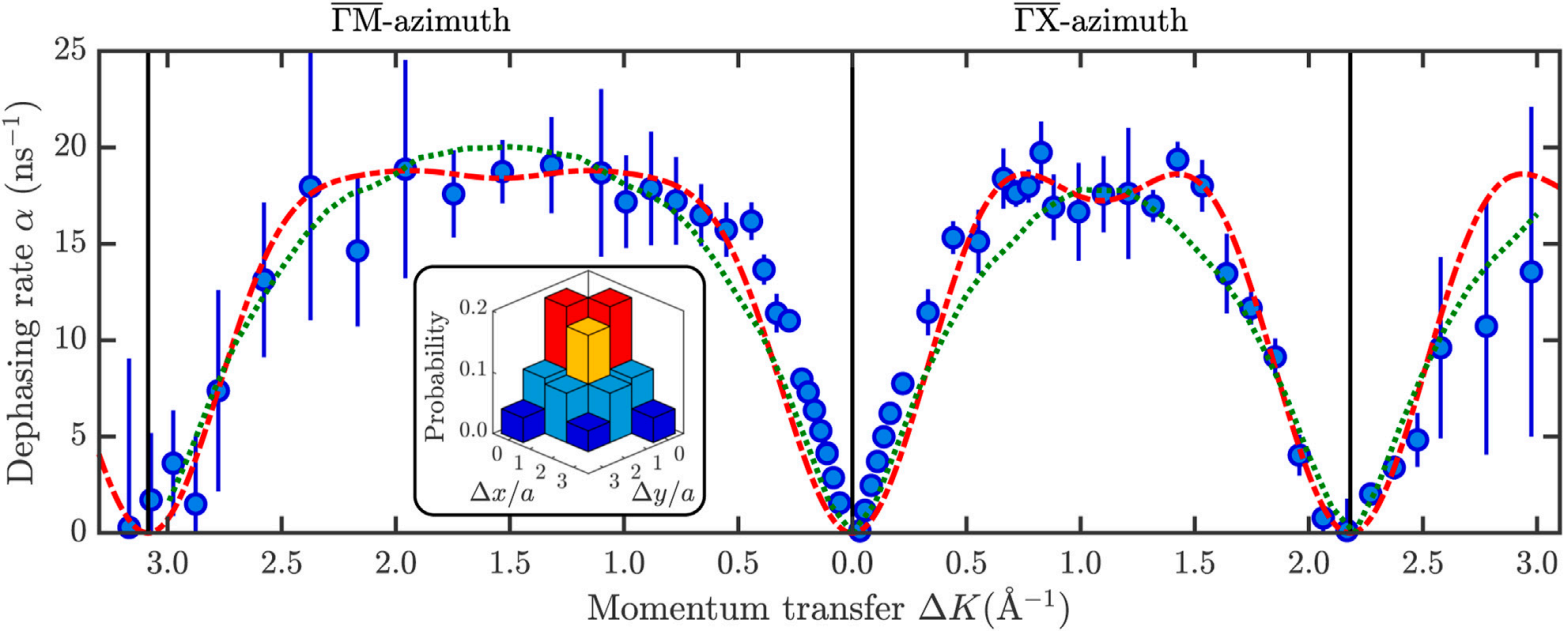
Momentum transfer dependence of *α*(Δ**K**) for the diffusion of CoPc on Ag(100) at 350 K and a CoPc coverage of 0.11 ML. The red dash-dotted line shows the Chudley-Elliott model (Eq. 2). The corresponding jump distribution for displacements Δ*x* and Δ*y* in units of the surface lattice constant *a*, as shown in the inset, illustrates that a significant number of long jumps is present. The green dotted line, according to molecular dynamics simulations, allows a quantitative assessment of the rate of energy transfer between the CoPc molecule and the substrate.

To determine *α*(Δ**K**) experimentally, HeSE measurements were acquired for a range of Δ**K** values along a selected azimuth by systematically varying the angle of incidence *ϑ*_*i*_ and extracting the dephasing rate *α* from the resulting exponential decays. The dependence of *α*(Δ*K*) on the momentum Δ*K* = |Δ**K**| for the diffusion of CoPc on Ag(100) (*T*_*S*_ = 350 K) is shown in Figure 2 for the two high-symmetry directions of the crystal. For the case that the diffusion of the adsorbate is governed by the interaction of the molecule with a corrugated surface, its motion can be well described by the Chudley-Elliott (CE) model of jump-diffusion (Chudley and Elliott, 1961; Barth, 2000; Jardine et al., 2009; Ferrando and Jardine, 2020). The CE model provides an analytic solution for *I*(Δ**K**, *t*) with the dephasing rate *α* vanishing when the momentum transfer Δ**K** matches a reciprocal surface lattice vector and varying sinusoidally in between. It assumes that a particle rests for a time *τ* at an adsorption site before it moves to another adsorption site. In the simplest case, this motion happens on a Bravais lattice and the dephasing rate *α*(Δ*K*) becomes:

$$\alpha \left(\Delta K\right)=\frac{2}{\tau }\underset{n}{\sum }p_{n}\sin^{2}\left(\frac{\Delta K\cdot l_{n}}{2}\right)$$ (2)

where **l**_*n*_ are the corresponding jump vectors and *p*_*n*_ is the relative probability that a jump to the corresponding site occurs.

Figure 2 shows that the experimental data is best fitted with a CE model (red dash-dotted line, (Eq. 2)) with jumps on the square Ag(100) lattice. The jump distribution is displayed in the inset of Figure 2 and shows a significant fraction of long jumps. The corresponding residence time *τ* is 0.11 ns with a jump frequency of about 4.6 GHz to one of the nearest neighbour sites. We see already from the significant number of long jumps at 350 K that there is a clear difference to the STM data of the same system by Antczak et al. (2015a) taken at 45–50 K where hardly any long jumps are present. Long jumps are a modification of the simple molecular diffusion picture where jumps occur solely between adjacent sites as mentioned in the introduction: The adsorbate can move across several lattice distances within a single jump, bypassing multiple adjacent sites. Similar to the presented jump distribution here, it was also shown for single-atom diffusion on metals that longer jumps need to be introduced to explain the jump distribution at higher temperatures (Senft and Ehrlich, 1995; Antczak and Ehrlich, 2004) and we will further discuss that in Section 3.4.

Based on the momentum transfer dependence in Figure 2, the CoPc molecule can either jump from hollow to hollow site or from top to top site since only jumps between these adsorption sites give rise to the characteristic minima as shown by the CE model. Bridge sites would give rise to jumps on a square lattice as well, however, the shorter jump vector (compared to jumps between hollow sites) cannot reproduce the momentum transfer dependence of *α*(Δ**K**) as the position of the minima would be further out. On the other hand, from previous combined STM and density functional theory (DFT) studies (Antczak et al., 2015a) it is known that the CoPc molecule adsorbs with the central Co atom located at the hollow site (Figure 1A). Hence we conclude that the hollow site is the favourable adsorption site. As shown by Antczak et al. (2015a), during diffusion the molecule moves over the bridge site while the top adsorption site is the least favourable site (Antczak et al., 2015a) and the HeSE data confirms exactly this type of motion, with the only exception that at 350 K a high number of multiple or long jumps is present in contrast to the STM data at low temperature. Following the description of atomic-scale motion with the molecule moving or hopping along the surface, the substrate provides the thermal energy for the motion and friction is a direct measure of the molecule-substrate coupling as the rate of energy transfer determines the molecular diffusivity (Barth, 2000; Ala-Nissila et al., 2002; Jardine et al., 2009)—as discussed in Sections 3.3 and 3.4.

We further note that close to |Δ**K**| = 0 the experimental data points tend to be slightly above the curve given by the CE model. Such a deviation could be due to adsorbate-adsorbate interactions, however, it is not clearly evident whether it is repeated around the position of the diffraction peaks (vertical lines at about $3\;{\overset{{}^{\circ}}{A}}^{-1}$ along $\bar{\Gamma M}$ and $2.2\;{\overset{{}^{\circ}}{A}}^{-1}$ along $\bar{\Gamma X}$ ). Hence it could also be due to a motion of the CoPc molecule perpendicular to the surface (Alexandrowicz and Jardine, 2007).

### 3.2 Adsorbate interactions and coverage dependence

**FIGURE 3:**
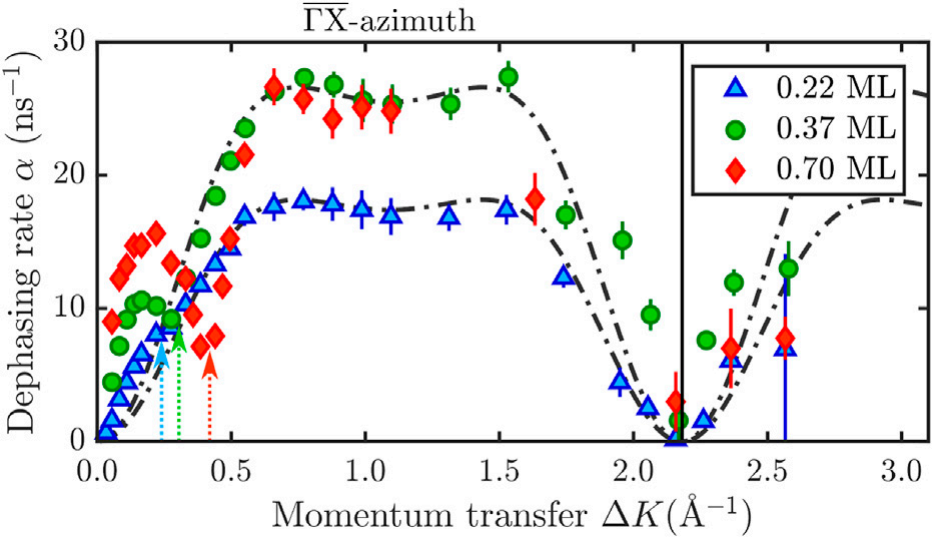
With increasing CoPc coverage, adsorbate interactions become evident in the experimental data giving rise to a distinctive peak and dip (illustrated by the arrow) structure in the *α*(Δ*K*) curve at low Δ*K* which becomes more significant with increasing coverage. The *α*(Δ*K*) curves are shown along $\bar{\Gamma X}$ for *T*_*S*_ = 350 K, with the grey dash-dotted lines from the CE model suggesting that the jump distribution remains the same while only the rate, i.e., amplitude of the model increases with coverage. The lowest coverage (0.11 ML, Figure 2) is not shown here since it is almost identical to the data taken at 0.22 ML.

Repulsive interactions become clearly evident at higher coverages, in particular close to the critical CoPc coverage (Antczak et al., 2017). While there is hardly any difference in the low coverage regimes (Figure 5B), the signature for adsorbate-interactions starts to set in above ≈1/4 ML, as shown in Figure 3. A significant change in the shape of the *α*(Δ*K*) curve can be seen as the coverage increases from 0.22 ML to 0.37 ML, and 0.7 ML. For the two lowest coverages (0.11 and 0.22 ML), the deviation from the CE model occurs only close to |Δ**K**| = 0 and is not repeated around the position of the diffraction peak, likely due to a motion of CoPc perpendicular to the surface (Alexandrowicz and Jardine, 2007).

With further increasing coverage, i.e., at 0.37 and 0.7 ML, the *α*(Δ*K*) in Figure 3 indicates that the CoPc dynamics is governed by correlated motion where the CoPc motion can no longer be described as isolated self-diffusion. The characteristic shape of the curve, featuring a peak at small Δ*K* values, followed by a de Gennes narrowing dip (Alexandrowicz and Jardine, 2007; Jardine et al., 2009) (illustrated by the arrows in Figure 3), has been predicted both numerically and analytically for surface diffusion of repulsive particles (Ala-Nissila et al., 2002; Jardine et al., 2009) but has only recently been observed experimentally (Alexandrowicz and Jardine, 2007; Tamtögl et al., 2021). The location of the dip corresponds to a peak in the static structure factor (Serra and Ferrando, 2002), verifies the repulsive nature of the force, and also allows a coverage estimation of the adsorbate (see 1.2 in the Supplementary Information).

While there are fewer data points available at higher coverage, Figure 3 clearly indicates that the jump distribution according to the analytic CE model (grey dash-dotted line) remains the same over the studied coverage regime. Outside the Δ*K* regions where the repulsive forces are present, the data points are still reproduced by the CE model with the same jump distribution as in Figure 2, the only difference being the overall rate, i.e., the amplitude of the analytic curve. We thus conclude that the jump distribution is not influenced by the coverage and is indeed a pure feature of the increased surface temperature as described in Section 3.3. We note that there seems to be hardly any change in the maximum rate when going from 0.37 ML to 0.7 ML while the position of the de-Gennes dip clearly wanders as expected. The latter may be correlated with the critical CoPc coverage observed at room temperature (Wagner et al., 2022a; b), i.e., where the CoPc molecules are in a 2D gas phase up to around 0.7–0.8 ML and the actual condensation of the 2D ordered structure occurs above 0.8 ML.

Before we attempt to directly compare activated diffusion results with STM measurements, these results show already that the combination of HeSE and STM measurements is extremely useful in investigating complex diffusion mechanisms, including also a large range of different coverages. It remains challenging to observe repulsive interactions with STM and the influence of repulsive forces is typically explored in conjunction with Monte Carlo methods (Trost et al., 1996). Here it was shown recently that repulsive interactions can be made visible by immobilising a fraction of molecules at step edges: Mobile molecules cannot reach the immobilised molecules, leaving behind a span of pristine silver between both due to the repulsive interaction (Antczak et al., 2017). Hence the HeSE measurements are complimentary to the STM measurements, as the former allow us to observe repulsive interactions “directly” during the diffusion process including also high coverage regimes, confirming the presence of repulsive interactions in the diffusion of CoPc on Ag(100) (Antczak et al., 2017).

### 3.3 Activated diffusion and mass transport at different time-scales

A direct comparison with the diffusivity of CoPc on Ag(100) obtained from real-space STM at low temperatures is easiest to achieve when plotting the HeSE rates in an Arrhenius plot analogous to the analysis of STM data. Therefore, temperature-dependent measurements at a fixed momentum transfer, Δ**K**, have been performed. For thermally activated processes, Arrhenius’ law predicts a temperature dependence of the dephasing rate, *α*, as:

$$\alpha =\alpha_{0}\;{\text{e}}^{-\frac{E_{a}}{k_{B}\;T_{S}}}$$ (3)

where *α*_0_ is the pre-exponential factor describing the jump attempt frequency, *E*_*a*_ is the activation energy for diffusion, *k*_*B*_ the Boltzmann constant and *T*_*S*_ the temperature of the sample surface. Taking the natural logarithm of (Eq. 3) results in a linear relationship between the inverse of the temperature, 1/*T*_*S*_, and the natural logarithm of the dephasing rate *α*.

**FIGURE 4:**
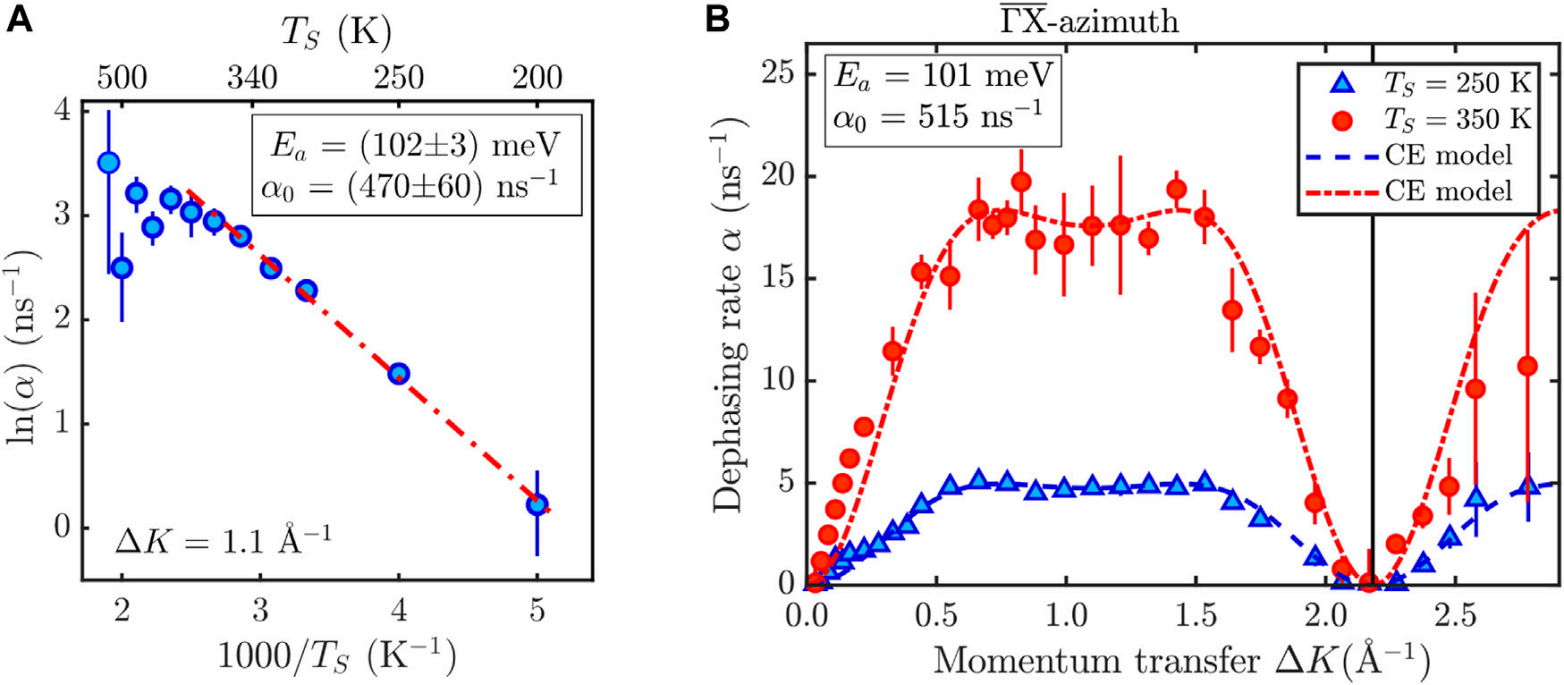
**(A)** Pre-exponential factor *α*_0_ and the activation energy *E*_*a*_ can be determined from an Arrhenius plot at a fixed momentum Δ*K* over a temperature range of 200 − 500 K. **(B)** Temperature dependence over the whole momentum transfer range along the $\bar{\Gamma X}$ -azimuth for a CoPc coverage of 0.11 ML. The dash-dotted and dashed lines show the corresponding CE model according to (2).

Figure 4A shows an Arrhenius plot obtained at a fixed momentum transfer Δ*K* over a wide temperature range from 250 K to about 500 K. We note that in general there is a deviation from the linear behaviour above ≈450 K which is likely to be due to the presence of multiple decays in the ISF. One possible explanation for the bending of the Arrhenius plot at higher temperatures is that with increasing temperature the activation of a metastable adsorption state occurs, located above the ground state with a different rotation of the molecule with respect to the substrate. The latter is, however, beyond the scope of the current study and will not be discussed in detail (Ellis et al., 2024).

The activation energy is then obtained from the slope of the linear fit in Figure 4 whereupon the intercept gives *α*_0_. For the linear fit and the determination of the activation energy, we have excluded the high-temperature points where an obvious deviation from the linear behaviour occurs. From Figure 4A we obtain *E*_*a*_ = (102 ± 3)meV and *α*_0_ = (470 ± 60)ns^−1^. The activation energy *E*_*a*_ is similar to the value obtained by STM measurements at low temperatures with 150 meV (Antczak et al., 2015a) but still significantly lower. It illustrates that while the microscopy measurements at 50 K are directly comparable with spin echo measurements in the higher 200–500 K temperature range, the diffusive motion undergoes subtle changes with increasing temperature.

In order to prove the consistency of the determined activation energy *E*_*a*_ across surface length-scales, we have also performed a measurement of the complete momentum transfer dependence along $\bar{\Gamma X}$ , at two different temperatures. Figure 4B shows the extracted *α*(Δ**K**) for 250 and 350 K, respectively. The shape of the CE model (dash-dotted and dashed line) is similar for both temperatures, indicating that the hopping motion, including a significant number of long jumps, remains comparable over this temperature range. At the same time, the hopping rate changes with temperature which is expressed in terms of the residence time *τ* in Eq. 2. We can use the change of the residence time *τ* with temperature to calculate again the activation energy which has been determined from the Arrhenius plots above. Here *τ* = 4 ⋅ 10^–10^ s at a sample temperature of 250 K and *τ* = 1.1 ⋅ 10^–10^ s at 350 K. This gives an activation energy of *E*_*a*_ = 101 meV which is consistent with the activation energy determined from the Arrhenius plots.

We can also compare the activation energy with the values found for CuPc on Ag(100) since the diffusion of both CoPc and CuPc on Ag(100) follow the same fashion (Antczak et al., 2015b). However, for the diffusion of CuPc on Ag(100), conflicting values regarding the activation were reported ranging from 30 to 81 meV measured at 140–220 K (Ikonomov et al., 2010; Hahne et al., 2013) and about 200 meV around 75 K (Antczak et al., 2015b). While the first two values were obtained from signal fluctuations of a locally fixed STM tip at higher substrate temperatures, our study illustrates that such a large variation of the activation energy is unlikely to be caused by the increased temperature.

We also note bond formation and clustering at higher temperatures for other Pc molecules in the literature. On the other hand, such an effect would only lead to an immobilising of several molecules and would hence give rise to a decreased number of diffusing molecules. It will thus not have any significant impact on the diffusion of the remaining mobile molecules and as such cannot explain the behaviour in the Arrhenius at high temperature (Ellis et al., 2024).

Finally, the diffusion coefficient *D* for two-dimensional motion, i.e., tracer-diffusion can be calculated from the hopping rate as determined from the CE model using:

$$D=\frac{1}{4}{\left\langle l\right\rangle }^{2}\Gamma$$ (4)

where Γ is the hopping rate and ⟨*l*⟩ the mean jump length (Barth, 2000; Jardine et al., 2009; Ferrando and Jardine, 2020). For a hopping rate of 1.9 ⋅ 10^10^ s^−1^ and a mean jump length of 4.52 Å we obtain a diffusion coefficient of:

$$D=9.6\cdot 10^{-10}\;{\text{m}}^{2}/\text{s},$$

at 350 K. *D* measured at 350 K corresponds to a prefactor for the diffusivity with *D*_0_ = 2.7 ⋅ 10^–8^ m^2^/s and is thus much smaller than *D*_0_ ≈ 1 ⋅ 10^–5^ m^2^/s as reported in STM works by Antczak et al. (2015a). While the latter may at first glance appear as a stark discrepancy, it can clearly be related to the reduced effective diffusion barrier *E*_*a*_ obtained at higher temperatures. It further illustrates that for a precise determination of any prefactor *D*_0_, measurements should ideally include also higher temperatures, closer to the extrapolation in the Arrhenius’ plot. Thus reports about the fast diffusion of large molecules such as decacyclene and hexa-tert-butyldecacyclene molecules on Cu(110) as observed in STM measurements (Schunack et al., 2002) should also be treated with care, in particular upon extrapolation of the diffusivity to high temperatures.

### 3.4 Long jumps and atomic-scale friction of CoPc

**FIGURE 5:**
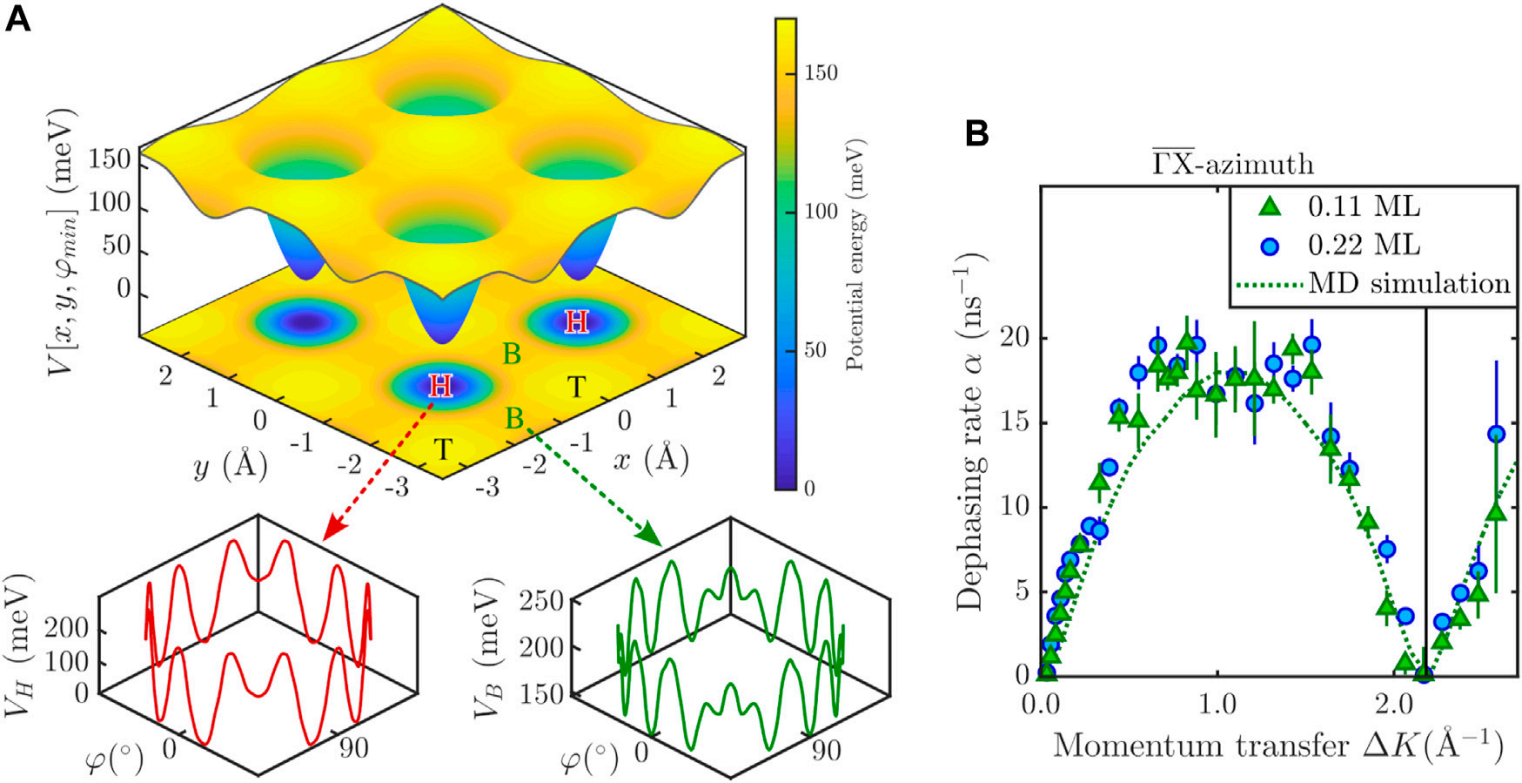
**(A)** Energy landscapes used in the MD Langevin simulations. The upper panels show the lateral potential energy surface (PES) *V* (*x*, *y*), for the minimum rotational energy configuration at each site. The color map extends from the lowest energy (hollow site, H) via the most favourable transition state (bridge site, B), and the highest potential energy site (top site, T). The lower panels show the potential energy as a function of rotation *φ* around the *C*_4_ molecular axis perpendicular to the surface: The red line on the left is the potential *V*_*H*_(*φ*) at the hollow site, while the green line on the right is the potential *V*_*B*_(*φ*) at the bridge site. **(B)** Comparison of the *α*(Δ*K*) curves for the two lowest CoPc coverages together with the green dotted line, according to the MD simulation.

As shown in the present example, long jumps can dominate high-temperature motion of large organic molecules. While the mechanism of long jumps has been characterised in the case of self-diffusion with field ion microscopy (FIM) on tungsten about 20 years ago (Antczak and Ehrlich, 2005; Antczak, 2006b; a; Antczak and Ehrlich, 2007), the mechanism has hardly been considered for more complex molecules. In the case of metal-atom diffusion following FIM, long jumps are seen as a continuation of single jumps: Detailed analysis of these jumps provides evidence that they still proceed via the lowest potential energy route, but the equilibration of the adatom proceeds in the second or third potential well (Figure 5A), in the case of a double or triple jump, respectively (Antczak and Ehrlich, 2007). As such, long jumps replace a single jump in the atomistic picture and while the total number of jumps follows an exponential increase with temperature, the occurrence of particular jump types does not. For single atom diffusion, long jumps become clearly more important as the temperature increases (Braun and Ferrando, 2002; Pollak and Miret-Artés, 2023) and it has also been suggested that these are a general feature of hydrogen diffusion on close-packed transition metal surfaces at high temperatures (Townsend and Avidor, 2019).

The observation that a large number of long jumps are present at high temperatures already suggests that despite its molecular size, CoPc exhibits a low friction *η* during diffusion on Ag(100). Moreover, as the analytic CE only tells us the “end” site where the molecule ends up but not the continuation of the molecule in a long jump, a common method to further analyse HeSE data often follows from molecular dynamics (MD) simulations based on solving the Langevin equation (Avidor et al., 2019; Sacchi and Tamtögl, 2023). The Langevin description of dynamics allows a quantitative analysis of the atomic-scale friction *η*, with *η* being a direct measure of the coupling between the molecular motion and the heat-bath of the substrate and thus determining the rate of barrier crossing.

In the MD simulations, the interaction of the adsorbed molecule with the substrate atoms is described by a “frozen” potential energy surface (PES, Figure 5A), where the friction coefficient *η* describes the rate of energy transfer between the molecule and the surface. As one may anticipate for a complex molecule such as CoPc a simple center-of-mass (CoM) MD cannot capture the full details of the diffusion process, however, as shown in Figure 5B the correct jump distribution is reproduced reasonably well with an MD simulation that includes the translational CoM motion and the rotation of the CoPc molecule, in analogy to the low-temperature STM findings by Antczak et al. (2015a).

The potential energy surface used in the simulation is derived from the DFT calculations given in Antczak et al. (2015a) with the exact details described in Molecular dynamics simulation in the Supplementary Material. As illustrated in Figure 5A, the potential consists of a translational part *V* (*x*, *y*) (top panel) and a rotational part, for rotations *φ* around the *C*_4_ molecular axis perpendicular to the surface (bottom panel, see also Hedgeland et al. (2016)). The latter varies with the adsorption site between hollow (H) sites given by *V*_*H*_(*φ*), and bridge (B) sites given by *V*_*B*_(*φ*).

A typical trajectory from an MD simulation with (*x*, *y*) and *φ* *versus* time is shown in the Supplementary Material (Supplementary Figure S2). The momentum transfer dependence *α*(Δ*K*) as extracted from the MD simulation is shown as a green dotted line in Figure 2 as well as in Figure 5B. By varying the friction *η* under the assumption that the rotational friction is the same as the translational one (see Section 1.3 in the Supplementary Material) we obtain a good fit with the experimental data for

$$\eta =0.23\;{ps}^{-1}.$$

To our knowledge, this is the first report of the atomic scale friction of such a large molecule (The molecular mass of CoPc being 571.46 u whereas previous studies of larger molecules included typically adsorbates with an atomic mass below 100 u) (Jardine et al., 2009; Hedgeland et al., 2016). Friction coefficients have been determined for a wide range of different systems, although most work is concentrated on small adsorbates and in the low-temperature region. The atomic-scale friction *η* for atomic adsorbates is typically smaller than 1 ps^−1^ (Jardine et al., 2009; Rittmeyer et al., 2016), and only for larger, organic molecules, friction values of e.g., *η* = 1.8 ps^−1^ for pentacene (C_22_H_14_) on Cu(110) have been reported (Rotter et al., 2016). On the other hand for benzene (C_6_H_6_) on Cu(001) a value *η* = 0.4 ps^−1^ was reported (Hedgeland et al., 2016) when considering both translations and rotations as in the present case. Hence, while one may anticipate increasing friction and thus lower diffusivity for larger molecules, the additional degrees of freedom may impose a different trend, i.e., the diffusivity becomes significantly greater than that of a point-like particle with the same mass. In other words, the observed diffusion rate is significantly higher if additional molecular degrees of freedom such as the rotation of the CoPc molecules are included, with the friction *η* = 0.23 ps^−1^ for rotations and translations of CoPc upon diffusion over Ag(100). At the same time, as *η* is the force divided by the mass and the CoPc mass with 571.46 u is very large, we are nevertheless still dealing with very large forces.

In summary, we note that a simple MD that considers both rotations and translations is thus useful to obtain a quantification of the energy transfer rate, even though it cannot capture the full details of the diffusion mechanism of a complex organic molecule over the entire temperature range (Ellis et al., 2024). The observation that an “effective” potential different from the simple geometry of substrate atoms is present is not new and has, e.g., already been observed in the case of atom self-diffusion (Oh et al., 2003)—however it has not been considered for large molecules where the molecular degrees of freedom give rise to a multi-dimensional potential energy surface thus contributing to the “effective” barrier during diffusion.

## 4 Discussion

We have studied the diffusion of CoPc on Ag(100) using helium spin-echo spectroscopy over a wide temperature and coverage range. Comparison of our data with STM measurements shows broad agreement for data taken at low temperature with STM (Antczak et al., 2015a) and data taken with HeSE in a seven times higher temperature regime. While the diffusion of CoPc still follows a similar route along the potential energy surface, with HeSE we can clearly distinguish the onset of multiple/long jumps with increasing temperature. We thus illustrate the mechanism of long jumps which has been previously characterised by FIM for self-diffusion of tungsten atoms, in the case of large molecular adsorbates on surfaces.

Moreover, the experimentally obtained activation energy for the diffusion of CoPc is significantly lower at high temperatures compared to the barrier obtained in previous STM measurements at cryogenic temperatures (Antczak et al., 2015a). The result illustrates that a precise determination of single-molecular diffusivity measurements and the prefactor *D*_0_ should ideally also include high-temperature data. Previous measurements extrapolating cryogenic STM data over the entire temperature scale and associated high diffusivities should thus be treated with care as mechanistic changes of the molecular motion which can only be observed at high temperatures may be present.

We have further determined the rate of energy transfer between the molecule and the substrate in terms of the atomic-scale friction with *η* = 0.23 ps^−1^. Thus, despite the large molecular size, CoPc/Ag(100) is clearly a low friction scenario and the diffusivity can be understood by including additional molecular degrees of freedom in terms of a combined rotational and translational motion.

Finally, we have illustrated that by the combination of two experimental techniques, HeSE with STM, it is possible to obtain a complete picture of complex diffusion mechanisms and energy dissipation in surface diffusion over the entire temperature range, from the onset of diffusion to technologically relevant temperatures. While STM is capable of providing information in the low-temperature regime, HeSE can be used to get a better insight into diffusion at higher temperatures as well as at high coverages including the occurrence of long jumps, and adsorbate interactions. The herein presented approach promises also to provide a route by which the factors affecting the underlying structural assembly in organic thin films can be explored and understood at a molecular level.

## Data Availability

The raw data supporting the conclusion of this article is available via: https://doi.org/10.3217/z5d3c-jev61.
