# Supplementary material for: Single-molecular diffusivity and long jumps of large organic molecules: CoPc on Ag(100)
Source: Front Chem. 2024 Feb 6;12:1355350. doi: 10.3389/fchem.2024.1355350 (PMC10876995; doi:10.3389/fchem.2024.1355350)
Supplement: Supplementary file 1 [file Presentation1.pdf]

Supplementary Information

1 ADDITIONAL EXPERIMENTAL AND COMPUTATIONAL DETAILS

1.1 Adsorption, uptake and coverage calibration

In order to calibrate the coverage and to investigate the adsorption of CoPc on Ag(100), the helium specular signal I was measured while dosing CoPc at surface temperatures of 250 and 350 K. Figure 1 shows the relative He specular peak height I/I_0 as a function of CoPc exposure. Surface exposure is defined as the impinging flux of CoPc on the surface integrated over the time of exposure. For the calculation of the exposure we used an ion gauge correction factor of 50 for CoPc. (There is no specific report for CoPc in the literature but in general this seems to be in the order of 50 based on the values reported for other large hydrocarbons).

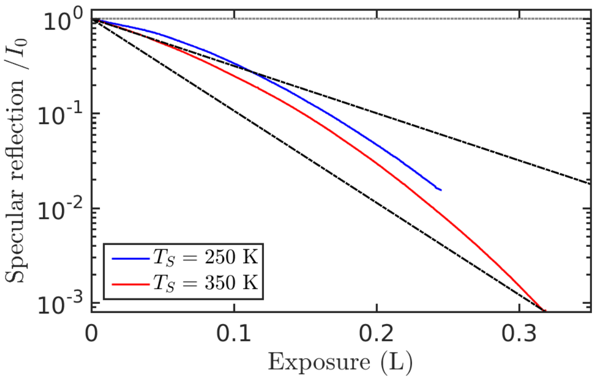

Figure 1a. Normalised specular helium-scattering signal I/I_0 as a function of CoPc exposure for an Ag(100) substrate temperature of 250 and 350 K. The dash-dotted lines represent the theoretical signal for a single exponential dependence versus exposure.

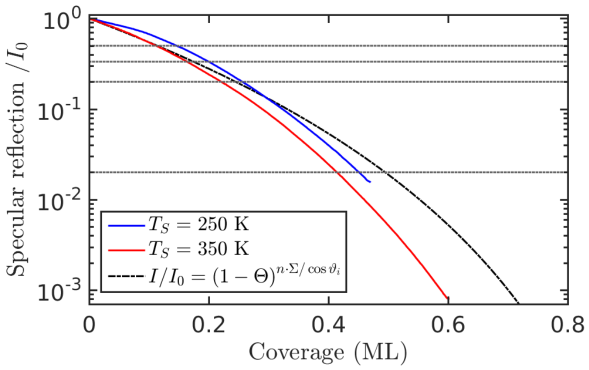

Figure 1b. I/I_0 versus coverage plotted on a logarithmic scale. The coverage has been determined from the exposure assuming an initial sticking coefficient of one. The dashed line corresponds to equation S1 with $\Sigma = 1000$ Å^2.

Figure 1. Uptake curves following the deposition of CoPc on Ag(100).

The fact that the presence of CoPc on the surface substantially attenuates the specular beam can be used as a measure of the CoPc coverage. The normalised specular intensity I/I_0 can be related to the CoPc coverage Θ via:

$$I/I_0 = (1-\Theta)^{n\cdot\Sigma/\cos\vartheta_i} \tag{S1}$$

$$\approx 1 - \Theta\cdot n\cdot\Sigma/\cos\vartheta_i \quad \text{for} \quad \Theta \ll 1 \tag{S2}$$

where n is the adsorbate density at monolayer (ML) coverage, Σ is the helium scattering cross section and the term $\cos(\vartheta_i)$ accounts for the increase of the apparent scattering cross section since scattering happens at an incident angle $\vartheta_i = 22.2°$. The adsorbate density n is given by the monolayer coverage. As described in the main part of the manuscript, monolayer coverage of CoPc corresponds to a (5×5) superstructure which is also commonly observed in low energy electron diffraction and STM investigations on Pc on Ag(100) (Salomon et al., 2013, 2015; Antczak et al., 2015a). As illustrated in Figure 1(a) of the main

text, CoPc adsorbs in a flat face-to-face orientation with an azimuthal rotation of about 30° around the C_4 molecular axis perpendicular to the surface. The van der Waals radius of the molecule along the diagonal molecular axis is $\approx 1.73\,\text{nm}$ meaning that an individual molecule covers approximately (5×5) surface unit distances.

In the low-coverage regime, a linear dependence of the intensity on the coverage can be assumed (equation S2) and the scattering cross section Σ can be determined from the initial slope of the adsorption curve. In our case we obtain a scattering cross section of $\Sigma \approx 1000\ \text{Å}^2$ for CoPc on Ag(100). This value is in agreement with a rough estimation of the CoPc form factor: based on an approximate description of the molecule with a cylindrical shape and a radius of ≈ 18 Å the interference pattern upon He scattering from isolated CoPc molecules can be described according to (Lahee et al., 1987).

The coverages as determined from the uptake curves are summarised in Table 1.

Table 1. CoPc coverages based on the uptake curve at 350 K: Coverage Θ for each specular attenuation I/I_0 where dynamics measurements have been performed.

I/I_0	Θ (ML)	ΔK_{dip} (Å^{-1})	Θ (ML)
1/2	0.11	-	-
1/5	0.22	0.24	0.22
1/50	0.45	0.3	0.37
1/30000	0.75	0.42	0.7

1.2 Coverage dependence and repulsive interactions

As described in the main part of the manuscript, with increasing coverage repulsive interactions between the individual CoPc molecules give rise to a characteristic shape of the α-ΔK curve (Figure 3), featuring a peak at small ΔK followed by a de Gennes narrowing dip. We can also use the position of the de-Gennes narrowing (dip) to estimate the coverage. In accordance to calculating a scaled (1×1) diffraction pattern in reciprocal space, the position of the de-Gennes dip is given by $\Delta K_{dip} = 4\pi/\sqrt{3}r$ where r is the preferred spacing among the adsorbates in this quasi-hexagonal structure. Using the (5×5) superstructure as monolayer coverage the coverage Θ is then given via:

$$\begin{aligned} \Theta &= \frac{75\, a^2\, \Delta K_{dip}^2}{16\pi^2} \\ &= 3.94 \cdot \Delta K_{dip}^2 \end{aligned} \tag{S3}$$

where a is the surface lattice constant of Ag(100) and ΔK_{dip} the momentum transfer at which the de-Gennes narrowing occurs. There appears a clear dip at the two highest coverages in Figure 3 which gives $\Theta = 0.37$ ML for $I_0/50$ and $\Theta = 0.7$ ML for $I_0/30000$. These values are slightly smaller than those obtained from the uptake curves but since (S3) is only an approximation based on a long-range quasi-hexagonal structure it shows that the estimation of the coverage is certainly consistent.

Finally, the overlap of giant cross sections with increasing coverage, enables also to obtain information about the adsorbate inter-molecular forces from the uptake curves since both repulsive and attractive forces have distinct signatures (Farías and Rieder, 1998). In the case of repulsive forces the scattering cross sections of the individual adsorbates overlap less, increasing the total cross section seen by the He beam

compared to that for non-interacting adsorbates and thus the uptake curve falls below the linear line which is clearly evident for the uptake curve taken at $350\,\mathrm{K}$ in Figure 1a.

1.3 Molecular dynamics simulation

As mentioned in the main text, while molecular dynamics (MD) simulations based on the Langevin approach cannot provide a complete description of the entire degrees of freedom, they are useful to obtain information about the atomic-scale friction η of the system, which directly influences the diffusion as it affects the rate of energy transfer between the adsorbate and the substrate. While for purely Brownian motion, η can be directly extracted this is not possible for hopping motion as in the present case and instead MD simulations are often employed. An MD simulation package which was implemented in Simulink and MATLAB based on the generalised Langevin equation (GLE), is available from https://doi.org/10.5281/zenodo.2025809 under the GNU/GPL-3.0 license and is described in detail in Avidor et al. (2019). We use the MD simulation as a minimalist computational experiment, with the same temperature as in the experiment ($350\,\mathrm{K}$), while the remaining parameter η describes the energy transfer and is adjusted so that the experimental data points are reproduced as shown by the green dotted line in Figure 2 as well as in Figure 5(b) of the main text.

In the MD simulations the motion parallel to the surface and the rotation of the molecule about a vertical axis are modelled, and the motion of the molecule motion is simulated by using the Langevin equation. The interaction with the substrate is given by a potential energy surface over which the molecule moves, a friction, characterised by the parameter, η, defined such that the frictional force is given by $\mathbf{F} = -\eta m \mathbf{v}$, and random noise impulses of mean magnitude as given by Einstein's theory of Brownian motion applied at each time step to maintain the mean temperature of the diffusing species.

The friction associated with the rotational motion, characterised by the frictional couple, G, the moment of inertia, I, about the axis normal to the surface, and the angular velocity, ω, ($G = -\eta I \omega$) is set up with the same parameter η as for the translational motion. While one might expect the frictional drag to increase with the size of a molecule, the mass increases also with the size of the molecule so friction-induced acceleration of each part of a large molecule will be comparable and thus also the angular acceleration for a large molecule such as CoPc. Hence the assumption that η is the same for both types of motion is reasonable in the absence of any additional information.

The potential energy surface used in the simulation is derived from the Density Functional Theory (DFT) calculations given in Antczak et al. (2015a) in which the rotational variation of the adsorption energy of the CoPc molecule on Ag(100) is reported for the hollow, $V_H(\varphi)$, and bridge, $V_B(\varphi)$, adsorption sites. As there are no calculations available for the top site, it is assumed that the top adsorption sites exhibit the same angular potential dependence as the bridge site, and the overall potential is defined as:

$$V(x,y,\varphi) = [1 - f(x,y)]\, V_H(\varphi) + f(x,y) V_B(\varphi) + V_{add}(x,y) \quad , \tag{S4}$$

with $V_{add}(x,y)$ according to Equation S6 and the interpolation function is given by:

$$f(x,y) = \frac{1}{4}\left[3 + \cos\left(\frac{2\pi x}{a}\right) + \cos\left(\frac{2\pi y}{a}\right) - \cos\left(\frac{2\pi x}{a}\right)\cdot\cos\left(\frac{2\pi y}{a}\right)\right] \quad . \tag{S5}$$

Equation S5 has the value 0 at the hollow site $(\frac{a}{2}, \frac{a}{2})$ where a is the Ag-Ag nearest neighbour distance in the substrate and 1 at the top and bridge sites. A detailed plot of the final overall PES is illustrated in Figure 5(a) in the main text.

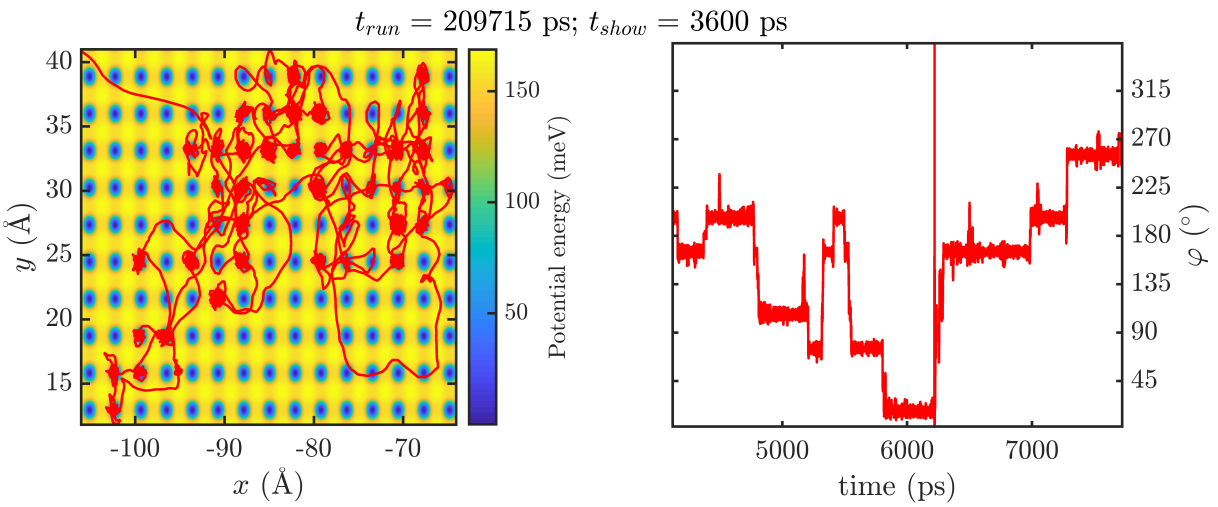

Figure S2. Detail of a typical trajectory from the Langevin molecular dynamics simulation. The left panel shows the (x, y) coordinate over a duration of 3600 ps, superimposed as red line on a contour plot of the lateral potential for the minimum rotational configuration. The right panel shows the molecular rotation φ as a function of time.

The potential is increased by 5.3% to make the minimum energy required to pass from one unit cell to the next 150 meV in line with the activation energy determined from the temperature dependence of the hopping rates in the STM study by Antczak et al. (2015a). The assumption that the measured activation energy should match the potential energy barrier at low temperatures is based on the fundamentals of transition state theory (Ellis et al., 2024). Moreover, the STM data shows jumps only over the bridge sites i.e. in the $[110]$ type directions, with no evidence above the noise in the data for diagonal jumps over the top site in the $[100]$ directions (Antczak et al., 2015a). Typical uncertainties in the STM data for the published Arrhenius plots are about $1/50^{\text{th}}$ of the jump numbers recorded, which puts an upper limit on the relative rate of jumps over the top and bridge site which, via a Boltzmann factor on a typical temperature of measurement (47 K), gives a lower limit on the increase in potential energy at the top site compared to the bridge site of 16 meV. A potential was therefore created which has a top site increased over the bridge site by the minimum amount required to be consistent with the STM data by adding the function:

$$V_{add}(x,y) = \frac{\Delta V_{TB}}{4}\left[1+\cos\left(\frac{2\pi x}{a}\right)+\cos\left(\frac{2\pi y}{a}\right)+\cos\left(\frac{2\pi x}{a}\right)\cdot\cos\left(\frac{2\pi y}{a}\right)\right] \quad , \tag{S6}$$

where the energy difference between top and bridge sites, $V_{TB} = 16$ meV is set to the minimum required by the STM data.

We further note, that a 2D centre of mass (CoM) Langevin simulation of the HeSE data can only be made to fit the data at 350 K with bridge and top site energies of 157 meV and 132 meV and a friction parameter of $0.3\,\text{ps}^{-1}$. However, having a top site energy that is less than that of the bridge site contradicts the requirements of the STM data as described above which shows only jumps over bridge sites. Hence, the higher molecular degrees of freedom during the motion are altering the jump distribution - as the molecule must be correctly aligned for the lowest energy at the transition state allowing the most likely “passage” through it. The most significant extra degree of freedom is the rotation φ about the C_4 rotational axis through the Co atom and perpendicular to the surface which is therefore included in the MD simulations.

To facilitate MD simulations for a further analysis of the experimental data, several MD simulations are run and the output of the MD simulations, the particle trajectories $\mathbf{R}(t)$, can then be used to calculate both

the coherent and the incoherent $I(\Delta\mathbf{K}, t)$ Avidor et al. (2019) which can further be analysed in the same manner as the experimental data. The detail of a typical trajectory as extracted from the MD simulation is plotted in Figure S2. The left panel shows the translational particle trajectory $\mathbf{R} = (x, y)$ superimposed as red line onto a filled contour plot representing the lateral PES for CoPc/Ag(100). The right panel shows the variation of the molecular rotation φ over time, here shown over a length of $3600\,$ps.
